# Spiral volumetric optoacoustic and ultrasound (SVOPUS) tomography of mice

**DOI:** 10.1016/j.pacs.2024.100659

**Published:** 2024-10-28

**Authors:** Sandeep Kumar Kalva, Ali Özbek, Michael Reiss, Xosé Luís Deán-Ben, Daniel Razansky

**Affiliations:** a Institute of Pharmacology and Toxicology and Institute for Biomedical Engineering, Faculty of Medicine, University of Zurich, Zurich CH-8057, Switzerland; b Institute for Biomedical Engineering, Department of Information Technology and Electrical Engineering, ETH Zurich, Zurich CH-8093, Switzerland; c Department of Biosciences and Bioengineering, Indian Institute of Technology Bombay, Mumbai 400076, India

**Keywords:** Optoacoustic imaging, Photoacoustics, Small animal imaging, Ultrasonography, Ultrasonic transducers

## Abstract

Optoacoustic (OA) tomography is a powerful noninvasive preclinical imaging tool enabling high resolution whole-body visualization of biodistribution and dynamics of molecular agents. The technique yet lacks endogenous soft-tissue contrast, which often hampers anatomical navigation. Herein, we devise spiral volumetric optoacoustic and ultrasound (SVOPUS) tomography for concurrent OA and pulse-echo ultrasound (US) imaging of whole mice. To this end, a spherical array transducer featuring a central curvilinear segment is employed. Full rotation of the array renders transverse US and OA views, while additional translation facilitates volumetric whole-body imaging with high spatial resolution down to 150 µm and 110 µm in the OA and US modes, respectively. OA imaging revealed blood-filled, vascular organs like heart, liver, spleen, kidneys, and surrounding vasculature, whilst complementary details of bones, lungs, and skin boundaries were provided by the US. The dual-modal capability of SVOPUS for label-free imaging of tissue morphology and function is poised to facilitate pharmacokinetic studies, disease monitoring, and image-guided therapies.

## 1 Introduction

Preclinical small animal imaging is essential in biomedical research to offer critical insights into disease progression and therapeutic effects [1], [2]. *In vivo* imaging of rodents is commonly achieved with downscaled versions of whole-body clinical modalities like X-ray computed tomography (CT) [3], [4], magnetic resonance imaging (MRI) [5], [6], or positron emission tomography (PET) [7], [8]. Ultrasound (US) imaging is also routinely used in preclinical studies as it provides good soft tissue contrast and real-time imaging capacity to assess blood perfusion and other biodynamics [9], [10]. Optoacoustic (OA) tomography is additionally gaining maturity as a preclinical imaging tool capitalizing on rich spectroscopic optical contrast to resolve the biodistribution and dynamics of absorbing molecules *in vivo* [11], [12], [13], [14], [15], [16]. State-of-the art OA tomography systems can reach imaging rates of hundreds to thousand frames per second dictated by the pulse repetition rate of the laser and excellent spatial resolution in the 20–200 µm range, mainly dependent on the detection bandwidth of the US transducer [17], [18], [19], [20]. Spiral volumetric optoacoustic tomography (SVOT) represents a particularly advantageous strategy for whole-body imaging of mice with scalable spatio-temporal resolution [21], [22], [23], [24], [25]. Since endogenous OA contrast is dominated by hemoglobin absorption [26], [27], [28], [29], accurate volumetric mapping of vascular anatomy and hemodynamics can be achieved in a label-free manner [30], [31]. However, accurate organ delineation and signal quantification is often compromised by the lack of soft tissue contrast.

Conversely, pulse-echo US offers a distinct advantage in obtaining soft-tissue information and is widely employed to differentiate morphological features relying on acoustic impedance mismatches [32], [33]. Both OA and US techniques share key advantages, such as the use of non-ionizing radiation, real-time imaging capacity, portability, or high spatial resolution. More importantly, both modalities rely on US detection, thus are natural imaging partners rendering complementary information. This has fostered the development of dual-modal OA-US imaging systems with unique capabilities for preclinical and clinical applications in early cancer diagnosis [34], [35], image guided surgeries [36], or therapy monitoring [37], to name a few examples. Hybridization of OA and US for dual-modal small animal imaging has been achieved with different types of US transducers, including spherical single-element sensors [38], [39], linear arrays [40], [41], [42], multi-segment arrays [43], [44], [45], or arc-shaped arrays [46], [47], [48]. These configurations often suffer from limited-view and out-of-plane artifacts when operating in cross-sectional (2D) imaging mode. Moreover, they require relatively long scan times, typically tens of minutes, to cover the entire animal. This hampers their use in studies involving pharmacokinetics and pharmacodynamics across large regions. On the other hand, spherical array transducers are growingly being used to achieve accurate tomographic OA reconstructions in real time [49], [50], [51], [52], [53], [54], [55]. However, compared to the sub-millimeter inter-element pitch used in typical clinical linear US transducer arrays, the large pitch of the spherical arrays (∼3 mm) hampers efficient acoustic beamforming for pulse-echo US imaging [56]. A fundamentally new strategy is then required to achieve concurrent volumetric OA and US imaging.

In this work, we introduce spiral volumetric optoacoustic and ultrasound (SVOPUS) tomography that employs a hybridized hemispherical array combining a cylindrically-focused, arc-shaped segment of emit/receive transducers and a dense grid of large square sensing elements distributed across the rest of the spherical surface. The proposed system thus achieves cross-sectional pulse-echo US imaging while ensuring optimal collection of volumetric OA data with a broad tomographic coverage. System's performance is quantified in terms of resolution and image quality in phantoms and further demonstrated with high-resolution dual-modal whole-body images of mice.

## 2 Materials and methods

### 2.1 SVOPUS tomography set-up

**Fig. 1:**
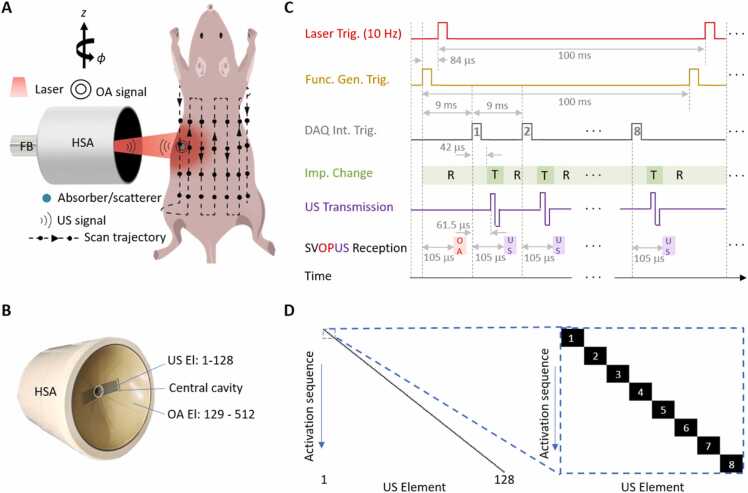
**The SVOPUS setup and imaging protocol. A.** Schematic representation of the SVOPUS scanner for whole-body volumetric imaging of mice. HSA: hybrid spherical array, FB: fiber bundle; OA: optoacoustic, US: ultrasound. **B.** Photo of the hybrid spherical array depicting the arrangement of OA and US elements. **C.** Time diagram of SVOPUS pulse transmission and signal acquisition sequence. T: Transmission, R: Reception. **D.** Activation sequence of the 128 US elements. Zoom-in shows the activation sequence of adjacent 8 elements after each laser pulse.

The schematics of the proposed hybrid SVOPUS scanner is depicted in Fig. 1A. An Nd:YAG laser (SpitLight, Innolas Laser GmbH, Krailing, Germany) delivering <10 ns pulses with 10 Hz repetition rate and 1064 nm optical wavelength was used as an OA signal excitation source. A custom-built fiber bundle (CeramOptec GmbH, Bonn, Germany), inserted into the central cavity of a custom-made hybrid spherical array (HSA, Imasonic Sas, Voray, France), was used to illuminate the mouse surface with an approximately Gaussian illumination profile of ∼10 mm diameter at full width at half maximum (FWHM). The optical fluence was maintained well below ANSI safety limits in all experiments [57]. SVOPUS image acquisition was performed with the custom-made HSA connected to multi-channel data acquisition/transmission unit (DAQ, Falkenstein Mikrosysteme GmbH, Taufkirchen, Germany), synchronized by the Q-switched laser trigger as detailed below. The HSA integrates a spherical matrix sub-array and a cylindrically-focused arc-shaped sub-array into a single device to render volumetric OA and pulse-echo B-mode US images, respectively (Fig. 1B). The spherical matrix sub-array segment consists of 384 elements arranged on a hemispherical surface having 40 mm radius and an angular coverage of 130^0^ (1.15π solid angle). The individual square-shaped elements have an approximate area of 12.20 mm^2^, inter-element pitch of 3.6 mm, and a central frequency of 5 MHz. The arc-shaped array segment consists of 128 elongated elements arranged at the central part of the HSA. The individual elements have 10 mm height, 0.2 mm inter-element pitch, and 10 MHz central frequency. All the elements of the HSA exhibit >50 % transmit/receive bandwidth at FWHM. The acquired OA and US signals were simultaneously digitized at 24 Megasamples per second with the DAQ and transferred through 1 Gb/s Ethernet connection to a computer. This is equipped with 128 GB random access memory and a NVIDIA GeForce GTX 1060 6 GB graphical processing unit, and was operated with Windows 10. Later, a workstation with arch Linux operating system, Intel i7–4820K (8) @ 3.9 GHz, 64 GB RAM, and a NVIDIA GeForce GTX TITAN X was used to process the signals and reconstruct the images.

### 2.2 SVOPUS scanning procedure

SVOPUS scanning was performed by step-and-go motion of the HSA together with the output of the fiber bundle along the vertical (z) and azimuthal (ϕ) directions (Fig. 1A). Specifically, full rotation (360°) over 18 angular positions and long-range displacement in 12 steps separated by 2 mm along the longitudinal axis to cover the mouse body was performed. Translation and rotation of the HSA was controlled using motorized stages (RCP2-RGD6c-I-56 P-4–150-P1-S-B, RCP2-RTCL-I-28 P-30–360-P1-N, IAI Inc., Shizuoka Prefecture, Japan).

At each position of the array, an emission/reception sequence for hybrid OA-US imaging was implemented as shown in Fig. 1C. A function generator (externally triggered with the Q-switch output of the previous laser pulse) was used to trigger the DAQ with 99.916 ms delay. The input impedance of the DAQ is then switched to reception mode. This external trigger delay to the DAQ was chosen such that the next laser excitation occurs 84 μs after each trigger event from the function generator with the generated OA signals falling within a 20 μs acquisition window delayed by 105 μs with respect to the external trigger. Note that the generated OA signals for the first laser pulse will not be acquired. The 100 ms time interval between the laser pulses was used for pulse-echo B-mode US imaging. The internal trigger of the DAQ was set such that a total of 8 US pulses at 9 ms intervals were transmitted and the reflected US waves were collected within this time window. In total, 16 laser pulses were needed to complete the US pulse-echo sequence with all 128 elements of the arc-shaped array segment. For each pulse-echo sequence, only one US element was used to transmit US pulse while all the 512 elements received the echoes with only 128 central (arc-shaped) array elements used for US beamforming. The US pulse transmission paradigm is shown in Fig. 1D. Each element of the arc-shaped array emits a bipolar square pulse with ±19 V_peak-to-peak_. The DAQ input impedance was altered during each US pulse at 42 μs after each internal trigger event and was subsequently changed back for corresponding echo signal reception after US pulse emission with 61.5 μs delay with respect to the internal trigger. These delays were chosen so that the pulse-echo US responses fall within a 20 μs acquisition window delayed 105 μs with respect to the internal trigger. Note that the DAQ continuously acquires the OA and pulse-echo US signals from all the 512 hybrid array elements for each trigger event. At each position of the HSA, a total of 108 volumetric OA and 6 cross-sectional US images were acquired and averaged, which lasts (108/10 Hz) = 10.8 sec. Hence, it takes (18*12*108/10 Hz) = 2332.8 sec ≈ 39 minutes to scan the whole body of the mouse.

### 2.3 System characterization

The spatial resolution of the SVOPUS system across the entire field of view (FOV) was characterized by imaging a phantom containing a cloud of 50 μm polyethylene microspheres (Cospheric Inc, Santa Barbara, USA). These microspheres were randomly distributed in a 20-mm diameter agar cylinder (1.3 % agar powder by weight). The sphere phantom was imaged following the step-and-go protocol described above with the HSA together with the fiber bundle scanned along 18 angular positions covering 360° and 3 translational positions along z-axis in 2 mm steps. The position of the HSA was controlled using motorized stages that can be translated in the vertical direction and rotated in the azimuthal direction. To improve accuracy, the acquired signals were averaged 100 times (OA mode) and 6 times (US mode) at each scanning position. The spatial resolution of the SVOPUS system was characterized by positioning the focus of the HSA at 5.7 mm distance from the rotation axis. Note that this distance was chosen to obtain relatively uniform spatial resolution across the entire FOV, as the spatial resolution is generally dependent on the position of the geometrical center of the spherical array relative to the axis of rotation [23]. The motor positions were controlled using a MATLAB-based interface. After each experiment, the relative position and orientation of the HSA with respect to its rotation axis were calibrated using a gauge phantom, which consisted of a single 200 μm polyethylene microsphere (Cospheric Inc, Santa Barbara, USA) embedded in a 20-mm diameter agar cylinder (1.3 % agar powder by weight). It was scanned to determine the radial position, lateral shift, and axial rotation angle of the HSA by considering 18 angular locations covering 360°.

### 2.4 *In vivo* animal experiments

Female Hsd:Athymic Nude-Foxn1nu/nu mice were used for the *in vivo* demonstration experiments, in accordance with the Swiss Federal Act on Animal Protection and with the approval of the Cantonal Veterinary Office in Zurich. The mice were anesthetized using isoflurane (5 % volume ratio for induction and 1.5 % volume ratio during experiments; Provet AG, Switzerland) in an oxygen/air mixture of 100/400 mL/min. The mice were held in a fixed position with fore and hind paws secured using a custom-made animal holder and placed inside a water tank. The water temperature was maintained at 36 °C throughout the experiments using a feedback-controlled heating stick. A vet ointment (Bepanthen, Bayer AG, Leverkusen, Germany) was applied on the eyes to prevent dehydration during scanning and for protection against laser light. The mice were scanned in a step-and-go fashion to cover 360° over 18 angular positions and in 12 steps separated by 2 mm in the vertical direction. At each position of the HSA, a total of 108 volumetric OA frames and 6 fully-compounded cross-sectional US frames were acquired and averaged in order to increase the signal-to-noise-ratio of the images.

### 2.5 Signal processing, image reconstruction, and analysis

The acquired time-resolved OA and US signals were initially band-pass filtered in the 0.1–15 MHz frequency band covering the entire bandwidth of the HSA elements. The OA images were reconstructed with the signals recorded with the 384 elements of the spherical sub-array segment whilst the 128 elements of the arc-shaped sub-array were solely used for the US reconstructions with conventional delay-and-sum beamforming technique implemented on a graphics processing unit (GPU) [58], [59], [60]. To facilitate *in vivo* OA image reconstruction, a pre-processing self-gated respiratory motion rejection algorithm was applied for the step-and-go scanning method, as described elsewhere [61]. Briefly, the recorded 108 raw OA data frames (1 frame per laser pulse), each frame containing 493 time-samples for every channel, were rearranged into a 2D matrix containing 493 ×384 rows and 108 columns. This 2D matrix represents the entire sequence of frames acquired for a single position of the HSA. The correlation matrix of all frames was then computed. Clustering of frames into two sets with high and low correlation coefficients was performed by employing the second order *k*-means method to the correlation coefficient matrix. The frames with high correlation coefficient were then selected and averaged with the volumetric OA images reconstructed using a GPU-based back-projection (BP) technique [62], [63]. Time taken for clustering breathing frames in case of OA imaging is 113.4 sec. To reduce image artifacts due to spatial undersampling, each detector was additionally split into 9 sub-elements [25]. All these steps were performed at each position of the HSA. For phantom images, we assumed an average speed of sound (SOS) of 1545 m/s, 25 μm voxel resolution for OA, and 10 μm pixel resolution for US. For *in vivo* imaging, we used instead 1535 m/s SOS, 50 μm voxel resolution for OA, and 12.5 μm pixel resolution for US. Individual reconstructed 3D volumes of the mouse at each position of the HSA were stitched using Icmax compounding technique to obtain whole-body volumetric mouse images [25]. The reconstruction time for an OA image volume at a single position of the HSA was 19.4 sec, whereas the total reconstruction time required to obtain whole-body OA image was 74.7 minutes. The US beamforming at a single position of the HSA takes 23.2 sec with the cross-sectional compounded images over 18 angular positions taking 6.9 minutes to reconstruct. Note that these reconstruction times were calculated for OA reconstructions with 50 µm pixel resolution and for US reconstructions with 12.5 µm resolution. The quality of OA images can potentially be further enhanced by employing model-based reconstruction algorithms at the expense of longer computation times [64], [65].

## 3 Results

### 3.1 Spatial resolution characterization

**Fig. 2:**
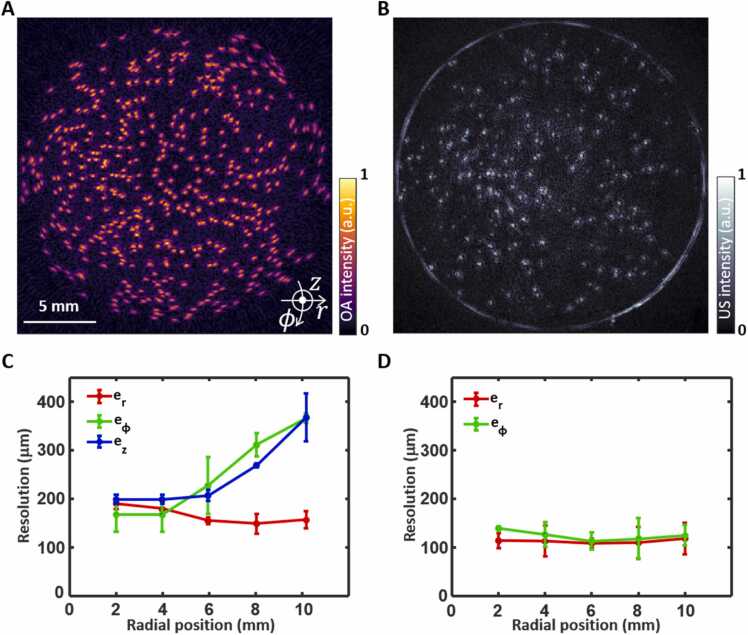
**Spatial resolution characterization of the SVOPUS system.** Reconstructed maximum intensity projection (MIP) OA **(A)** and US **(B)** images of the 50 μm microspheres phantom. The focus of the hybrid spherical array geometry was set at a distance of 5.7 mm from the axis of rotation. **C.** Dependence of radial ($e_{r}$), azimuthal ($e_{\phi }$), and elevational ($e_{z}$) resolution on the radial position from the axis of rotation for OA imaging. **D.** Dependence of radial ($e_{r}$) and azimuthal ($e_{\phi }$) resolution on the radial position from the axis of rotation for pulse-echo US imaging.

Results of the spatial resolution characterization of the SVOPUS system are shown in Figs. 2A and 2B. For volumetric OA imaging, the spatial resolution along three cylindrical axes, namely, radial $e_{r}$, azimuthal $e_{\phi }$, and elevational $e_{z}$ directions were computed, whilst only in-plane components ($e_{r}$ and $e_{\phi }$) were considered for the B-mode US imaging (Figs. 2C and 2D). The spatial resolutions were estimated at each radial position after deconvolving the actual microsphere diameter *D* from the corresponding FWHM in the images as $\sqrt{{\left(\mathrm{FWHM}\right)}^{2}-\;{\left(D\right)}^{2}}$. Note that FWHM was calculated after fitting to Gaussian curves for all axes. In both imaging modes the radial resolution performance remained nearly isotropic and constant throughout the imaged volume in the range between 150 μm and 190 μm for OA imaging and almost constant resolution around 110 μm for US imaging. Whilst strong variation of azimuthal resolution exists on the radial position for OA imaging ranging from 165 μm to 365 μm, slight variation exists for pulse-echo US imaging, ranging from 113 μm to 139 μm. The elevational resolution for OA imaging ranges from 200 μm to 370 μm. The spatially-dependent resolution in OA mode can be attributed to the directivity of the relatively large OA sensing elements towards the center of the FOV, in contrast to the wide angular sensitivity of high-pitch US sensing elements. Higher in-plane (radial and azimuthal) resolutions for pulse-echo US imaging were observed due to the higher central frequency of 10 MHz for the 128 elements of the arc-shaped array segment compared to that of 5 MHz for the remaining spherical array segment.

### 3.2 Whole-body mouse imaging

**Fig. 3:**
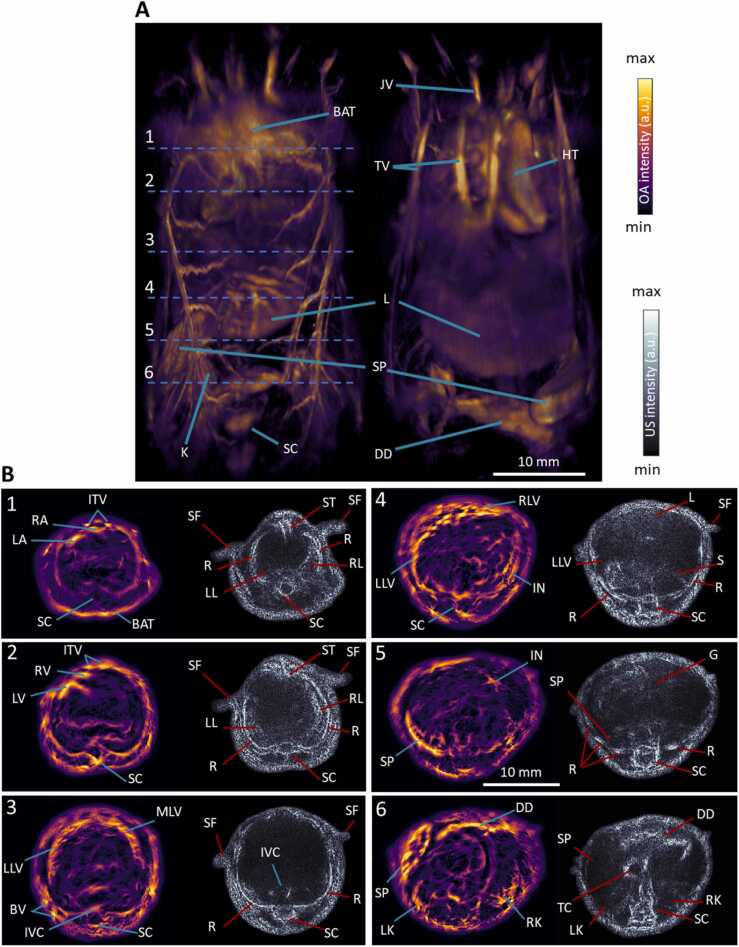
**Whole-body*****in vivo*****mouse imaging with SVOPUS. A.** Maximum intensity projections (MIPs) of the rendered volume (left to right) from the back and front views. Scalebar: 1 cm. **B.** OA and US cross-sectional slices at anatomical locations marked with dashed lines in **A**. BAT: brown adipose tissue, SC: spinal cord, SP: spleen, L: liver, K: kidney, HT: heart, JV: jugular vein, TV: thoracic veins, DD: duodenum, ITV: internal thoracic veins, RA: right atrium, LA: left atrium, RV: right ventricle, LV: left ventricle, ST: sternum, SF: skin folds, R: ribs, RL: right lung, LL: left lung, MLV: median lobe of liver, LLV: left lobe of liver, BV: blood vessels, RLV: right lobe of liver, IVC: inferior vena cava, IN: intestines, G: gut, TC: transverse colon, RK: right kidney, LK: left kidney.

Whole-body *in vivo* imaging performance of the hybrid SVOPUS system was subsequently demonstrated (Fig. 3). SVOT imaging of the entire mouse was obtained by stitching individual reconstructed volumetric frames at each position of the HSA by employing the Icmax compounding technique [25] (Fig. 3A). Major organs like the heart, liver, spleen, kidney, spinal cord, brown adipose tissue, duodenum and surrounding vasculature can be easily discernible in the maximum intensity projections (MIPs) along different views. The full tomographic angular coverage of the SVOPUS scanning method enables visualization of deep anatomical structures that are mainly concealed in the MIP views that mainly emphasize superficial signals due to depth-dependent light attenuation in tissue. The cross-sectional OA images (Fig. 3B, 0.1 mm thickness) at different anatomical locations (marked in Fig. 3A) depict more anatomical details across the mouse body. Fine anatomical and vascular structures such as the heart atria and ventricles, inferior vena cava, and liver lobes are clearly discernible. Additional anatomical information is obtained from the cross-sectional pulse-echo US images (Fig. 3B). Various bone structures such as sternum, ribs, spinal cord, air filled lungs, duodenum, transverse colon, and skin appeared as hyperechoic, whilst highly vascularized organs like liver, spleen, kidneys appeared as hypoechoic in the US images. Other structures, like stomach and gut, produce diffuse reflections and appear as gray or hypoechoic contrast. Note that all OA cross-sectional images were normalized with the estimated fluence [66] and further processed with a weighted (10 %) Frangi filter, while a modified Bessel function [67] was employed for time-gain compensation of the US images in order to increase their contrast across all depths.

## 4 Discussion and conclusions

The newly-developed hybrid SVOPUS tomography system offers complementary dual-modal contrast, allowing for comprehensive visualization of both soft tissues and surrounding vascular networks across the entire mouse body. This stands in contrast to the existing whole-body SVOT imaging systems, which excel at angiographic imaging but lack other soft-tissue information. The US array employed in SVOPUS was designed *ad hoc* to provide a large angular coverage for accurate OA tomographic reconstructions, whilst additionally integrating an arc-shaped array segment for efficient pulse-echo US imaging. US imaging was interleaved between consecutive laser pulses, where US transmission and laser pulses were precisely synchronized for the OA responses and US echoes to fall within the same acquisition window. The SVOPUS system achieves isotropic resolution at the center of FOV down to 150 μm and an in-plane resolution down to 110 μm in the OA and US modes, respectively. Complimentary morphological information of various bones like sternum, ribs, spinal cord, air filled lungs, gas filled stomach, gut, intestines, and skin boundaries was discernible with pulse-echo US imaging. On the other hand, blood-filled and highly vascularized organs, like heart, liver, spleen, kidneys, brown adipose tissue, intestines, and surrounding vasculature was best visualized with OA imaging.

A number of hybrid OA and US imaging systems based on linear arrays, linear-concave arrays, or arc-shaped arrays have previously been proposed for small animal imaging [38], [39], [40], [41], [42], [43], [44], [46], [47], [48]. While such configurations achieve high quality cross-sectional US images, they are sub-optimal for achieving large angular coverage as required for high quality 3D whole-body OA imaging of mice. As a result, cross-sectional imaging systems cannot provide accurate images of arbitrarily-oriented vascular networks. This can partially be compensated by employing Frangi-based (vesselness) filtering, albeit at the expense of introducing vessel-like networks that do not represent actual structures in the mouse [68]. In contrast, by employing truly 3D tomographic acquisition geometry the SVOPUS approach is advantageous for accurate visualization of vascular networks extending in all three dimensions. Multi-spectral imaging can additionally be performed in the OA mode by rapid tuning of the laser wavelength [22], [31]. This allows for visualizing oxygen saturation (sO_2_) in healthy and diseased tissues by unmixing oxy-hemoglobin (HbO) and deoxy-hemoglobin (HbR) content.

In summary, SVOPUS optimally combines the complementary contrast mechanisms of OA and US imaging into a single hybrid modality. This represents a significant advancement in the field of preclinical imaging addressing limitations of state-of-the-art implementations. The method enhances the visualization of soft tissues in a non-invasive manner and achieves label-free imaging of the organ parenchyma along with surrounding vascular networks. The dual-modal performance of SVOPUS sets a new standard for non-invasive imaging performance at the whole-body scale thus opening new avenues for preclinical studies into pharmacokinetics, monitoring of disease progression, and therapy guidance.

## CRediT authorship contribution statement

**Sandeep Kumar Kalva:** Writing – review & editing, Writing – original draft, Visualization, Validation, Software, Methodology, Investigation, Formal analysis, Data curation, Conceptualization. **Daniel Razansky:** Writing – review & editing, Supervision, Resources, Project administration, Methodology, Funding acquisition, Conceptualization. **Xosé Luís Deán-Ben:** Writing – review & editing, Software, Methodology, Formal analysis, Conceptualization. **Michael Reiss:** Investigation. **Ali Özbek:** Software.

## Declaration of Competing Interest

The authors declare that they have no known competing financial interests or personal relationships that could have appeared to influence the work reported in this paper.

## Data Availability

Data will be made available on request.
